# Supplementary material for: The Mesenchymal Niche in Myelodysplastic Syndromes
Source: Diagnostics (Basel). 2022 Jul 5;12(7):1639. doi: 10.3390/diagnostics12071639 (PMC9320414; doi:10.3390/diagnostics12071639)
Supplement: Supplementary file 1 [file diagnostics-12-01639-s001.zip › diagnostics-1779656-supplementary.pdf]

Supplemental data :

Type	Dysplastic lineages	Cytopenias	Ring sideroblasts in erythhroid elements of BM	Blasts	Cytogenetics
**MDS-SLD**	1	1 or 2	RS < 15%, (or <5%[2])	PB <1%<br>BM <5%<br>No Auer rods	Any, unless fulfills criteria for isolated del(5q)
**MDS-MLD**	2 or 3	1 to 3	RS < 15%, (or <5%[2])	PB <1%<br>BM <5%<br>No Auer rods	Any, unless fulfills criteria for isolated del(5q)
**MDS-RS**<br>**MDS-RS-SLD**	1	1 or 2	RS≧15%<br>(or ≧5%[2])	PB <1%<br>BM <5%<br>No Auer rods	Any, unless fulfills criteria for isolated del(5q)
**MDS-RS-MLD**	2 or 3	1 to 3	RS≧15% (or ≧5%[2])	PB <1%<br>BM <5%<br>No Auer rods	Any, unless fulfills criteria for isolated del(5q)
**MDS with isolated del(5q)**	1 to 3	1 to 2	None or any	PB <1%<br>BM <5%<br>No Auer rods	del(5q) alone or with 1 additional abnormality except -7 or del(7q)
**MDS-EB-1**	0 to 3	1 to 3	None or any	PB 2~4% or BM 5~9%, no Auer rods	Any
**MDS-EB-2**	0 to 3	1 to 3	None or any	PB 5~19% or BM 10%~19% or Auer Any	Any
**MDS-U With 1% PB blast**	1 to 3	1 to 3	None or any	PB=1%[3], BM	Any
**with SLD and pancytopenia**	1	3	None or any	PB <1%<br>BM <5%<br>No Auer rods	Any

Table S1. 2016 WHO Criteria of classifications of myelodysplastic syndromes. WHO: World Health Organization; MDS: myelodysplastic syndromes; PB: peripheral blood; BM: bone marrow; RS: ring sideroblasts; MDS-SLD: MDS with single lineage dysplasia; MDS-MLD: MDS with multilineage dysplasia; MDS-EB: MDS with excess blasts; MDS-U: MSD, [2] with SF3B1 mutation; [3] 1% PB blasts must be recorded on at least two separate observations .
